# Apigenin attenuates ischemia–reperfusion-induced pulmonary ferroptosis and fibrosis by activating the Nrf2/HO-1/GPX4 axis in mice

**DOI:** 10.55730/1300-0152.2732

**Published:** 2024-10-18

**Authors:** Liang ZHANG, Haojie LI, Shichen XU, Hao WEN, Chaoxiao YU

**Affiliations:** 1 Department of Respiratory and Critical Care Medicine, Yantaishan Hospital, Yantai, Shandong, China; 2 Department of Respiratory and Critical Care Medicine, Yantai Municipal Laiyang Central Hospital, Yantai, Shandong, China; 3 Department of Endocrinology, Haiyang People’s Hospital, Yantai, Shangdong, China; 4 Second Clinical Medical College, Binzhou Medical University, Yantai, Shandong, China

**Keywords:** Apigenin, lung ischemia-reperfusion injury, Nrf2/HO-1/GPX4, ferroptosis, fibrosis

## Abstract

**Background/aim:**

Acute lung injury (ALI) is a major cause of morbidity and mortality after lung ischemia-reperfusion injury (LIRI). In recent years, pulmonary ferroptosis and its associated fibrosis have been recognized as important causes of LIRI. The purpose of this study is to investigate apigenin (APG) as a potential therapeutic target for treating LIRI-induced pulmonary ferroptosis and fibrosis.

**Materials and methods:**

A rat model of LIRI was established and the rats were randomly divided into three groups, a sham group, a LIRI group, and an APG group. The pathological changes of the lung tissue were evaluated using hematoxylin-eosin staining and Masson’s trichrome staining. Alterations in lung function were assessed using the pulmonary permeability index, myeloperoxidase, and wet-to-dry weight ratio. The pulmonary ferroptosis levels were evaluated by testing Fe^2+^, the ratio of reduced glutathione to oxidized glutathione disulfide, and malondialdehyde. Western blotting was performed to investigate the effect of APG on the expression of ferroptosis and fibrosis biomarkers in the lung tissues.

**Results:**

The results show that APG pretreatment relieves LIRI-induced pulmonary pathological damage and functional abnormalities in rats. In addition, APG administration can significantly improve LIRI-induced pulmonary ferroptosis and fibrosis levels. However, using Nrf2 inhibitors to block the Nrf2/HO-1/GPX4 pathway significantly reversed these therapeutic effects.

**Conclusion:**

These findings suggest that APG protects against LIRI-induced ferroptosis and fibrosis of lung tissues via the activation of the Nrf2/HO-1/GPX4 axis.

## 1. Introduction

Lung ischemia-reperfusion injury (LIRI) is a frequent occurrence in clinical settings such as single-lung ventilation, pulmonary embolism lung transplantation, and cardiopulmonary bypass, and is associated with a high morbidity and death (Laubach and Sharma, 2016; Zhang et al., 2023). The etiology of LIRI is complex and includes several pathways and pathophysiological processes, such as inflammation, oxidative stress, and autophagy (Ferrari and Andrade, 2015; Chen-Yoshikawa, 2021). Recent research has discovered that LIRI-activated ferroptosis is a significant cause of inflammation, which exacerbates lung damage (Liu et al., 2023; Lu et al., 2023).

Ferroptosis is a novel type of cell death that is closely related to oxidative stress and is characterized by reactive oxygen species (ROS) production and lipid peroxidation (Stockwell et al., 2017). Ferroptosis is characterized by three hallmarks: lipid peroxidation, ferric divalent ion (Fe^2+^) accumulation, and abnormal glutathione (GSH) metabolism (Jiang et al., 2021). Ferroptosis inhibition has also been linked to several essential genes associated with the lipid peroxide elimination system, including glutathione peroxidase 4 (GPX4) and nuclear factor erythroid 2-related factor 2 (Nrf2) (Seibt et al., 2019; Anandhan et al., 2023). According to reports, ferroptosis contributes to LIRI by escalating the inflammatory response, oxidative stress, and tissue iron concentration (Dong et al., 2020; Li et al., 2020; Liu et al., 2023). However, the exact involvement of ferroptosis in LIRI requires further investigation.

**Figure 1:**
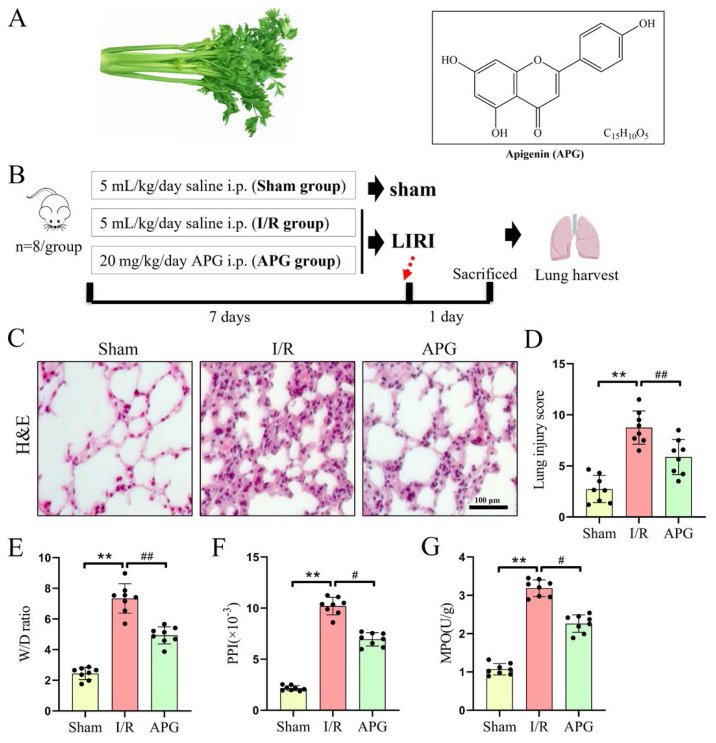
APG attenuated LIRI-induced lung lesions in rats. (A) A typical form and the chemical structure of APG. (B) A schematic of the experimental design. (C) Representative photomicrographs of the HE-stained lung sections for the three experimental groups. (D) Lung injury scores. (E) W/D ratios. (F) PPI values. (G) MPO activity. All data are expressed as mean ± standard deviation. ** = p < 0.01 for the sham and LIRI groups; # = p < 0.05 and ## = p < 0.01 for the LIRI and APG groups.

Apigenin (4′,5,7-trihydroxyflavone, APG) (Figure 1A) is one of the most abundant flavonoids found in plants, including thyme, celery, chamomile, and onions (Salehi et al., 2019). Numerous studies have found that APG has a variety of intriguing pharmacological features and potential applications in nutraceuticals (Salehi et al., 2019; Jin et al., 2022; Lee et al., 2023). For instance, its well-known antioxidant properties can be employed as a therapeutic agent to treat inflammation, autoimmune diseases, and cancer (Madunić et al., 2018; Xu et al., 2021). APG has been shown in recent research to inhibit nuclear factor-kappaB (NF-κB) activation and inflammation, potentially alleviating LIRI (Bougioukas et al., 2018). Evidence also suggests that APG exerts a protective effect against Di(2-ethylhexyl)phthalate (DEHP) induced iron death by activating GPX4 and inhibiting intracellular iron accumulation (Han et al., 2022). However, the repairing effects and potential mechanisms of APG on LIRI-induced pulmonary ferroptosis have yet to be understood.

This study was designed to investigate whether APG can alleviate LIRI-induced lung injury, particularly oxidative stress, fibrosis, and ferroptosis, and identify its potential mechanisms. This information could provide new strategies and a stronger theoretical basis for the application of APG in LIRI treatment.

## 2. Materials and methods

### 2.1. Animals and ethics statement

Male Wistar rats (200–250 g) aged 8–9 weeks were obtained from the SLAC Laboratory Animal Co., Ltd. (Shanghai, China) and housed in an SPF laboratory under conditions of standard humidity (40%–60%), temperature (25 °C), light/dark cycle (12 h), pellet diet, and water. The project was approved by the ethics committee of Binzhou Medical University (approval no. 2023-32).

### 2.2. Establishment of the LIRI model

The LIRI rat model was created using a previously defined technique (Ding et al., 2021). The rats were thoroughly anesthetized with 50 mg/kg pentobarbital sodium intraperitoneally (i.p.) before being placed on a homeothermic table to maintain core body temperature at 37 °C. A noninvasive artery clip was used to clamp the left pulmonary hilum, which resulted in total ischemia and hypoxia of the left lung for 1 h. The left lung’s ventilation and perfusion were then restored over the 2 h after the vascular clamp was loosened.

APG was obtained from MedChemExpress (#HY-N1201), and a stock solution of 5 mM was prepared in dimethyl sulfoxide (DMSO, #D2650, Sigma-Aldrich). The rats were divided randomly into three equal groups (n = 8). In the sham group, the rats underwent the identical thoracotomy operation but were not given a hilar block. In the APG group, the rats were subjected to LIRI and were administered APG (20 mg/kg/day) by intraperitoneal injection for 7 days before the LIRI model was established. In the LIRI group, the rats were subjected to LIRI and were given the same volume of 0.9% NaCl solution. Another group of eight rats received both APG (20 mg/kg/day) and Nrf2 inhibitor ML385 (30 mg/kg/day, i.p.) injections before the LIRI model was established.

For all groups, analgesics and anesthetics were used to minimize the suffering of the rats during the surgery. 24 h after the LIRI procedure, the rats were anesthetized with pentobarbital sodium (40 mg/kg, i.p.), blood samples were collected from the eye sockets, and they were euthanized by cervical dislocation. The rats were confirmed dead when there was no autonomous respiration, no reflex activity, and no heart activity.

The left lungs and blood samples were taken for further examination. A schematic diagram of groupings and interventions is shown in Figure 1B.

### 2.3. Staining

The center of each left lung was promptly fixed in 10% formalin and stored at 4 °C for 24 h. The tissues were then dehydrated, embedded in paraffin, sectioned at 5 μm, and stained with Masson’s trichrome (5 min, room temperature) and hematoxylin-eosin (HE) (5 min, room temperature). A microscope was used to obtain the images (Leica DM4000, Wetzlar, Germany). ImageJ (NIH) was used to calculate fibrosis as the percentage of the blue collagen-stained area relative to the total tissue in one field. All samples were assessed blind by two independent investigators (Li et al., 2018; Li et al., 2024).

### 2.4. Detection of lung tissue weight ratio

The initial wet weight was calculated by weighing freshly extracted left upper lung lobe samples. Then, the lung tissue samples were dried until reaching a consistent weight, and the dry weight was established. The wet-to-dry (W/D) weight ratio of the lung tissue was calculated by dividing the wet weight by the dry weight.

### 2.5. Determination of the pulmonary permeability index

The pulmonary permeability index (PPI) was measured as previously reported (Li et al., 2019). To determine total plasma protein, plasma supernatants were obtained and stored at −70 °C. 1 mL of normal saline was used for bronchoalveolar lavage of the lung. The bronchoalveolar lavage fluid (BALF) was centrifuged at 2000 rpm for 15 m. The BALF supernatant was then stored at −70 °C for the Bradford protein concentration assay to detect the total BALF protein concentration. The PPI was calculated by dividing the protein concentration of the BALF by the protein concentration of the plasma.

### 2.6. Detection of myeloperoxidase activity

To determine the myeloperoxidase (MPO) activity, 100 mg of lung tissue was mixed with 1 mL of RIPA lysate, homogenized with a glass homogenizer, placed on ice for 30 min to ensure complete cell lysis, and then centrifuged at 12,000 rpm for 15 min to collect the supernatants. The lung tissue MPO activity was measured using a colorimetry assay kit (#A044-1-1, Nanjing Jiancheng Bioengineering Institute) following the manufacturer’s instructions.

### 2.7. Fe^2+^, GSH/GSSG ratio, and lipid peroxidation assays

The relative iron concentration of the cell lysates was assessed with an iron assay kit (#ab83366, Abcam, UK). The levels of reduced glutathione (GSH) and oxidized glutathione disulfide (GSSG) were measured using a GSH/GSSG ratio detection assay kit (#ab205811, Abcam, UK). The relative concentration of malondialdehyde (MDA) in the cell lysates was assessed with a lipid peroxidation assay kit (#ab118970, Abcam, UK). All kits were used according to the manufacturer’s instructions.

### 2.8. Western blot analysis

The total protein was extracted from the lung tissues. The concentrations were detected using a Bradford protein assay kit (cat. no. P0006; Beyotime Institute of Biotechnology). The Western blot analyses were performed as described previously (Wang et al., 2022; Xiong et al., 2024a). After being blocked in 5% bovine serum albumin (cat. no. SW3015, Solarbio, China) and dissolved in tris-buffered saline with Tween-20 (TBST) (20% Tween 20) for 2 h at room temperature, the polyvinylidene fluoride membranes were then treated overnight at 4 °C with primary antibodies against Nrf2 (1:3000; #12721), heme oxygenase 1 (HO-1) (1:3000; #43966), GPX4 (1:3000; #52455), α-SMA (1:3000; #19245), FN(1:3000; #26836), COL-I (1:3000; # 72026), and β-actin (1:5000; #4970), all acquired from CST (Massachusetts, USA). The membranes were then incubated with a secondary antibody (1:5000; anti-rabbit IgG (H+L), #14708, CST, Massachusetts, USA) for 2 h at room temperature and then washed with TBST. ImageJ v.1.53 (National Institutes of Health, Rockville, MD) gel analysis software was used for the densitometric analysis.

### 2.9. Statistical analysis

Statistical analyses were carried out using SPSS v.19.0. Three independent experiments are represented as means ± standard deviation (Xiong et al., 2024b). A one-way analysis of variance with Tukey’s post hoc test and the unpaired Student’s t-test were used for comparisons between groups. Significance was established at p < 0.05.

## 3. Results

### 3.1. APG attenuated LIRI-induced lung lesions in rats

The effect of APG on lung injury following LIRI was assessed using HE staining for lung histology (Figure 1C). Notably, the LIRI group had disordered alveolar structure, considerable pulmonary interstitial edema, and a high number of inflammatory cells in the alveolar cavity. However, pretreatment with APG considerably reduced the LIRI damage histologically. Histological scores confirmed all of these alterations (Figure 1D).

Increased lung permeability aids the development of pulmonary edema. Thus, W/D ratio and PPI were used to determine the degree of pulmonary edema. In comparison to the sham group, the W/D ratios (2.44 ± 0.39 vs. 7.34 ± 0.96) and PPI values (2.16 ± 0.25 vs. 10.21 ± 0.86 × 10^−3^) significantly rose following LIRI, as seen in Figures 1E–1F. However, the APG pretreatment given to LIRI rats markedly reduced PPI (10.21 ± 0.86 vs. 6.94 ± 0.65 × 10^−3^) and W/D ratio (7.34 ± 0.96 vs. 4.93 ± 0.56). To assess neutrophil accumulation in the lung tissue, MPO activity was measured. Again, the APG pretreatment considerably decreased MPO activity (1.07 ± 0.15 vs. 3.19 ± 0.22 U/g), but LIRI significantly enhanced MPO activity (3.19 ± 0.22 vs. 2.26 ± 0.23 U/g), as Figure 1G illustrates. The results showed that APG pretreatment reduced LIRI-induced pulmonary damage in rats.

### 3.2. APG attenuated LIRI-induced pulmonary fibrosis in rats

**Figure 2:**
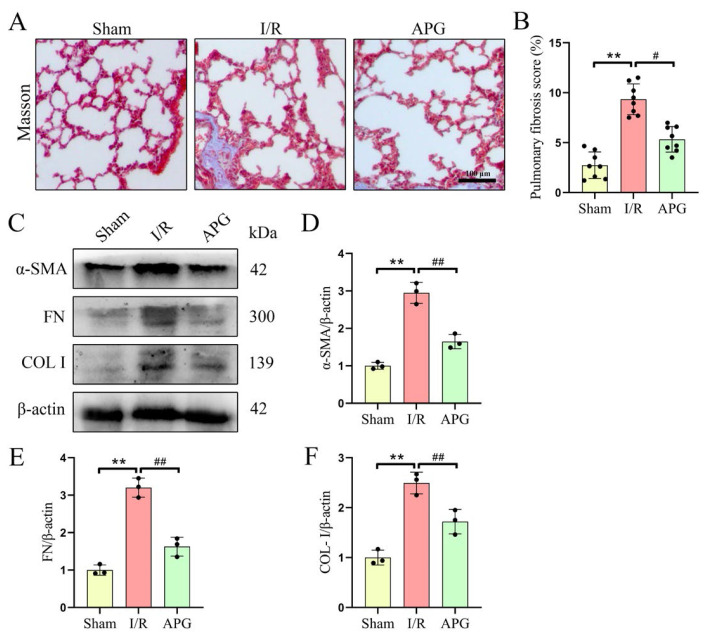
APG attenuated LIRI-induced pulmonary fibrosis in rats. (A) Representative photomicrographs of Masson’s trichrome-stained lung sections for the three experimental groups. (B) Pulmonary fibrosis scores. (C) Representative Western blot bands of the fibrosis-related protein expression levels. (D,E,F) Relative protein expression levels of α-SMA, FN, and COL-I, respectively. All data are expressed as mean ± standard deviation. ** = p < 0.01 for the sham and LIRI groups; # = p < 0.05 and ## = p < 0.01 for the LIRI and APG groups.

The effect of APG pretreatment on pulmonary fibrosis in LIRI rats was analyzed. Masson’s staining revealed that levels of pulmonary fibrosis in the LIRI group were markedly higher than those in the sham group (2.73 ± 1.33 vs. 9.34 ± 1.54 %) (Figures 2A and 2B). However, APG treatment significantly reduced the area of pulmonary fibrosis in LIRI rats (9.34 ± 1.54 vs. 5.33 ± 1.28 %) (Figures 2A and 2B). In addition, APG pretreatment significantly reduced the expression of the fibrosis-related molecules α-SMA (2.95 ± 0.28 vs. 1.65 ± 0.19), FN (3.20 ± 0.26 vs. 1.63 ± 0.25), and COL-I (2.49 ± 0.22 vs. 1.72 ± 0.22) in the lung tissues of LIRI rats (Figures 2C–2F). These results indicate that APG pretreatment significantly reduces fibrosis in the lung tissue of LIRI rats.

### 3.3. APG alleviates LIRI-induced pulmonary ferroptosis in rats

**Figure 3:**
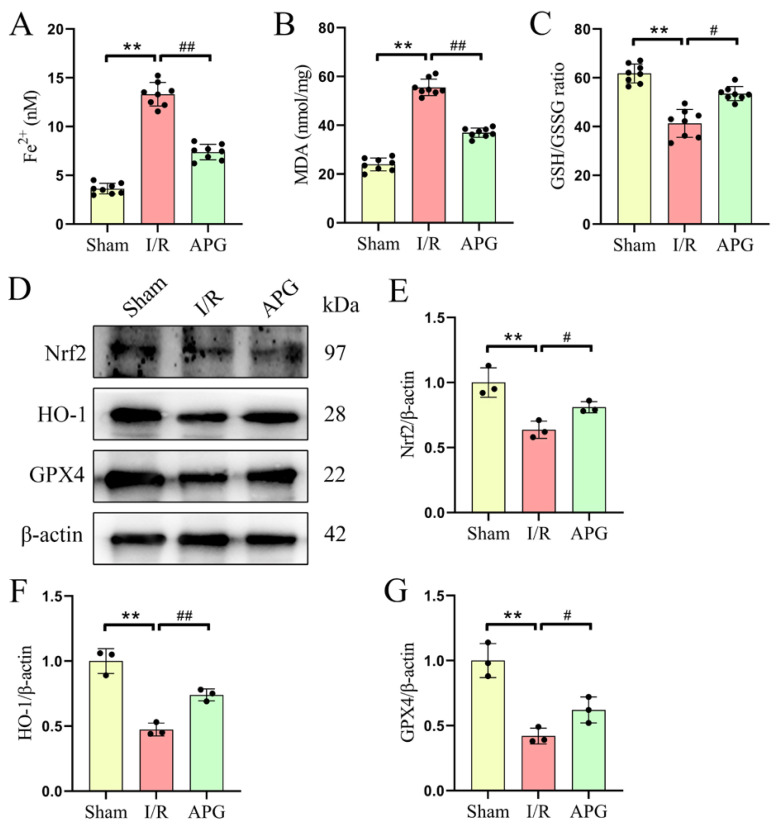
APG attenuated LIRI-induced pulmonary ferroptosis in rats. (A) Fe^2+^ levels. (B) MDA levels. (C) GSH/GSSH ratios. (D) Representative Western blot bands of ferroptosis-related protein expression levels. (E,F,G) Relative protein expression levels of Nrf2, HO-1, and GPX4, respectively. All data are expressed as mean ± standard deviation. ** = p < 0.01 for the sham and LIRI groups; # = p < 0.05 and ## = p < 0.01 for the LIRI and APG groups.

The ferroptosis level in the rat lung tissue was determined via the detection of the ferroptosis indicators Fe^2+^, MDA, and the GSH/GSSG ratio. As shown in Figures 3A–3C, the LIRI group had significantly higher MDA (23.94 ± 2.63 vs. 55.52 ± 3.38) and Fe^2+^ (3.63 ± 0.54 vs. 13.32 ± 1.22) levels and a significantly lower GSH/GSSG ratio (61.76 ± 3.89 vs. 41.29 ± 5.73) than the sham group. Pretreatment with APG significantly lowered MDA (55.52 ± 3.38 vs. 36.95 ± 1.90) and Fe^2+^ (13.32 ± 1.22 vs. 7.38 ± 0.80) levels and improved the GSH/GSSG ratio (41.29 ± 5.73 vs. 53.52 ± 2.90) in the lung tissues of LIRI rats (Figures 3A–3C).

Furthermore, the expression of Nrf2, a critical regulator of ferroptosis and oxidative stress, as well as the downstream antioxidant molecules HO-1 and GPX4, was markedly downregulated in the lung tissue of LIRI rats (Figures 3D–3G). Conversely, in the lung tissues of LIRI rats, administration of APG markedly increased the expression of Nrf2 (0.64 ± 0.07 vs. 0.81 ± 0.04), HO-1 (0.47 ± 0.05 vs. 0.74 ± 0.05), and GPX4 (0.42 ± 0.06 vs. 0.62 ± 0.10) (Figures 3D–3G). These data suggested that APG treatment reduces LIRI-induced pulmonary ferroptosis in rats.

### 3.4. Nrf2 blocking nullifies APG treatment of pulmonary ferroptosis in LIRI rats

**Figure 4:**
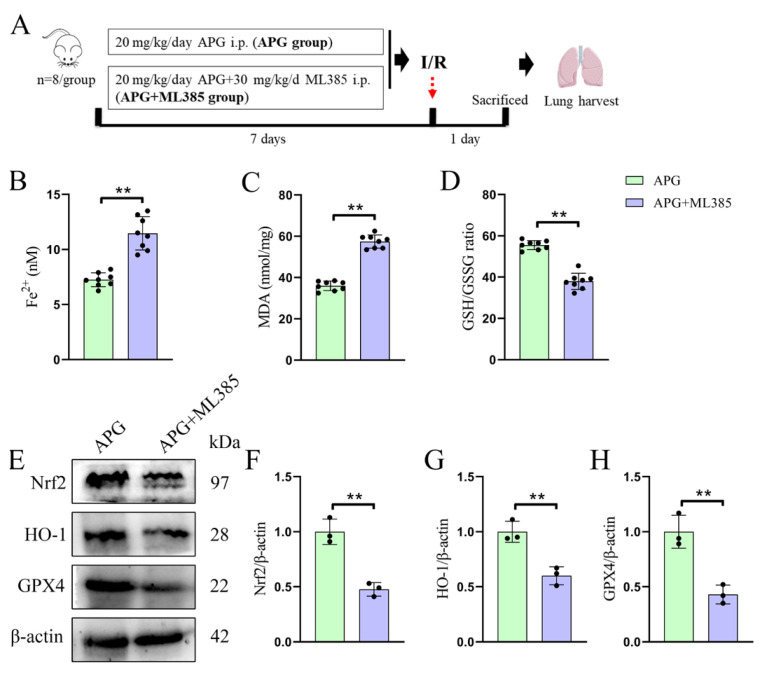
Blocking of Nrf2 nullifies APG treatment of pulmonary ferroptosis in LIRI rats. (A) A schematic of the experimental design. (B) Fe^2+^ levels. (C) MDA levels. (D) GSH/GSSH ratio. (E) Representative Western blot bands of ferroptosis-related protein expression levels. (F,G,H) Relative protein expression levels of Nrf2, HO-1, and GPX4, respectively. All data are expressed as mean ± standard deviation. ** = p < 0.01 for the APG and APG+ML385 groups.

To further establish the critical role of Nrf2 in the APG therapy for pulmonary ferroptosis and fibrosis of LIRI rats, ML385, an Nrf2 inhibitor was injected intraperitoneally 30 min before the APG injection daily (Figure 4A). As shown in Figures 4B–4D, the ML385 administration significantly increased MDA (36.00 ± 2.27 vs. 57.45 ± 3.19) and Fe^2+^ levels (7.25 ± 0.63 vs. 11.47 ± 1.51) in the APG-pretreated LIRI rats and decreased the GSH/GSSG ratio (55.55 ± 2.18 vs. 37.94 ± 3.94) in the lung tissues. Furthermore, the application of ML385 nullified the effects of APG on Nrf2 (1.00 ± 0.16 vs. 0.48 ± 0.06), HO-1 (1.00 ± 0.10 vs. 0.60 ± 0.08), and GPX4 (1.00 ± 0.15 vs. 0.43 ± 0.09) (Figures 4E–4H) in the lung tissues of LIRI rats.

### 3.5. Nrf2 blocking nullifies the therapeutic effect of APG on pulmonary fibrosis and pathological damage in LIRI rats

**Figure 5:**
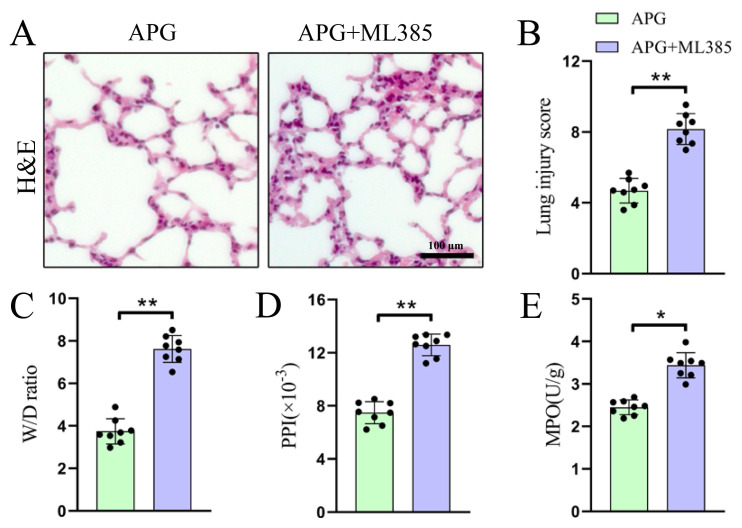
Blocking of Nrf2 nullifies the therapeutic effect of APG on pulmonary lesions in LIRI rats. (A) Representative photomicrographs of HE-stained lung sections. (B) Quantification of lung injury scores. (C,D,E) W/D ratio, PPI, and MPO activity, respectively. All data are expressed as mean ± standard deviation. * = p < 0.05 and ** = p < 0.01 for the APG and APG+ML385 groups.

The effects of blocking Nrf2 on the pulmonary fibrosis and pathological parameters in APG-pretreated LIRI rats was examined. As shown in Figures 5A–5B, the ML385 administration significantly increased lung injury scores (4.68 ± 0.69 vs. 0.8.17 ± 0.87) in the APG-pretreated LIRI rats. Furthermore, ML385 administration reversed the repair effect of APG treatment on the lung function indicators of W/D ratio (3.74 ± 0.60 vs. 7.62 ± 0.63), PPI (7.48 ± 0.83 vs. 12.59 ± 0.82 × 10^−3^), and MPO activity (2.45 ± 0.17 vs. 3.44 ± 0.30 U/g) in the LIRI rats (Figures 5C–5E).

**Figure 6:**
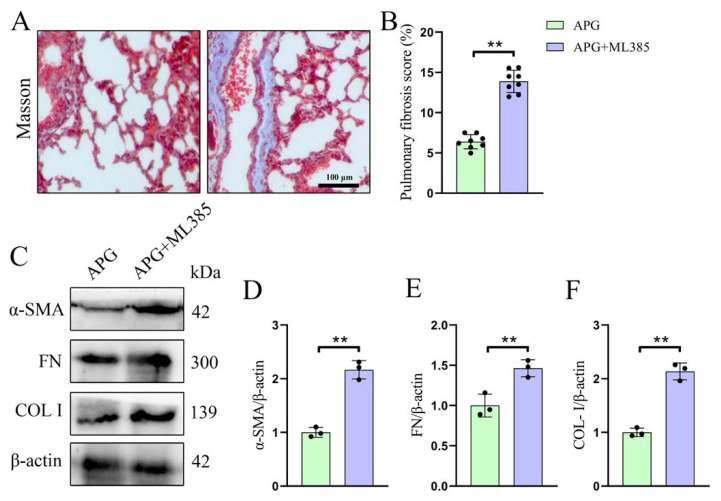
Blocking of Nrf2 eliminates the therapeutic effect of APG on pulmonary fibrosis in LIRI rats. (A) Representative photomicrographs of Masson’s trichrome-stained lung sections. (B) Pulmonary fibrosis score. (C) Representative Western blot bands of fibrosis-related protein expression levels. (D,E,F) Relative protein expression levels of α-SMA, FN, and COL-I, respectively. All data are expressed as mean ± standard deviation. ** = p < 0.01 for the APG and APG+ML385 groups.

Additionally, ML385 reversed the repair effect of APG treatment on pulmonary fibrosis in LIRI rats, significantly increased the fibrotic area (6.39 ± 0.88 vs. 13.89 ± 1.39 %) and the expression of fibrosis-related molecules α-SMA (1.00 ± 0.09 vs. 2.17 ± 0.17), FN (1.00 ± 0.14 vs. 1.46 ± 0.11), and COL-I (1.00 ± 0.08 vs. 2.14 ± 0.16) in the lung tissues of LIRI rats (Figure 6). These results indicate that APG pretreatment significantly reduces fibrosis in the lung tissue of LIRI rats and that blocking Nrf2 can reverse the APG repair effect on pulmonary fibrosis and pathological damage in LIRI rats.

## 4. Discussion

After LIRI, acute lung injury is the main cause of lung morbidity and death (Wang et al., 2023a). However, the exact mechanism of APG in LIRI is still being established. This investigation showed that in LIRI rats, APG can mitigate lung pathological damage, lessen pulmonary fibrosis, and restore lung function. Further research demonstrated that Nrf2/HO-1/GPX4 axis activation and ferroptosis blocking are crucial for APG to have therapeutic benefits for LIRI.

LIRI frequently occurs in a variety of clinical procedures and is usually accompanied by severe and devastating consequences leading to tissue damage (Laubach and Sharma, 2016). Severe forms can lead to primary graft dysfunction, which is a leading cause of short- and long-term morbidity and mortality after lung transplantation (Yang et al., 2022a). Numerous studies and our previous research have found that LIRI is associated with increased pathological damage and dysfunction in lung tissue (Zhang et al., 2021; Yang et al., 2022b; Zhang et al., 2023). Similarly, this study found significantly higher pathological injury scores and abnormal lung function markers (PPI, MPO, and W/D ratio) in LIRI rats. These abnormal lung tissue structures and functions can be drastically repaired with the injection of APG. According to reports, LIRI is accompanied by an increase in pulmonary fibrosis, and alleviating pulmonary fibrosis is also key to repairing LIRI-induced pulmonary dysfunction (Cao et al., 2021; Yang et al., 2022b). In this study, we found that LIRI rats had significantly higher levels of lung fibrosis and elevated expression of fibrosis-related markers (α-SMA, FN, and COL-I). Significantly, fibrosis damage can be reversed with APG treatment.

Pulmonary fibrosis is characterized by the accumulation of excessive connective tissue in the lungs, often accompanied by inflammation and oxidative stress (Koudstaal et al., 2023). Ferroptosis has drawn more interest lately as a possible treatment target for pulmonary fibrosis (Wang et al., 2023b). Studies have demonstrated that fibrotic lungs exhibit elevated levels of iron and iron-related proteins, suggesting a disturbance in iron homeostasis (Yue et al., 2023). Aberrant iron excess has been linked to lung fibrosis and functional decline in the bleomycin-induced pulmonary fibrosis mouse model (Ali et al., 2020). This current study also revealed a significant increase in the accumulation of Fe^2+^ and lipid peroxidation levels, while APG intervention significantly inhibited the occurrence of iron accumulation and lipid peroxidation in the lungs of LIRI rats. These findings suggest that APG blocking the process of ferroptosis may be a potential mechanism to alleviate pulmonary fibrosis and dysfunction during LIRI.

The signaling cascades and molecular targets implicated in ferroptosis, which can affect lipid peroxidation, antioxidant defense mechanisms, reactive oxygen species biochemistry, and iron metabolism, have been extensively researched (Jiang et al., 2021; Wang et al., 2023b). Nrf2 is an important transcription factor that helps cells survive oxidative stress, and its preservation is one of the primary strategies for avoiding ferroptosis. According to research, a drop in Nrf2 levels during ferroptosis exacerbates oxidative stress and leads to cell death (Dodson et al., 2019). Furthermore, Nrf2 activation-regulated antioxidant genes, such as HO-1 and GPX4, have drawn a lot of interest due to their significance in maintaining iron homeostasis and redox equilibrium. Aspalathin was reported by Jiang et al. to improve the Nrf2/HO-1 pathway, thereby attenuating glutamate-induced ferroptosis in HT-22 cells (Jiang et al., 2020). Ma et al. (2020) reported that melatonin reduces the level of ferroptosis in type 2 diabetes patients with osteoporosis by activating the Nrf2/HO-1 pathway. Yang et al. (2022c) found that Maresin1 prevented ferroptosis-induced liver damage by decreasing ROS generation and Nrf2/HO-1/GPX4 activation. The current study demonstrated that pretreatment with APG significantly boosts the Nrf2/HO-1/GPX4 pathway in the lung tissues of LIRI rats. Conversely, when ML385 was used to suppress Nrf2 activation, the therapeutic effect of APG on ferroptosis, fibrosis, and pulmonary dysfunction in LIRI rats was greatly reduced. These results suggest that the relief of LIRI-induced lung ferroptosis and fibrosis by APG is dependent on the activation of the Nrf2/HO-1/GPX4 pathway. This specific molecular mechanism remains to be further studied.

## 5. Conclusions

This study provides evidence that APG prevents LIRI-induced ferroptosis and fibrosis of lung tissues through the upregulation of the Nrf2/HO-1/GPX4 axis. These findings may bring fresh insights to the development of therapeutic applications of APG for LIRI.

## Data Availability

The datasets used and/or analyzed in this study are available from the corresponding author upon reasonable request.
